# The effect of helminth infection on the microbial composition and structure of the caprine abomasal microbiome

**DOI:** 10.1038/srep20606

**Published:** 2016-02-08

**Authors:** Robert W. Li, Weizhong Li, Jiajie Sun, Peng Yu, Ransom L. Baldwin, Joseph F. Urban

**Affiliations:** 1 United States Department of Agriculture, Agriculture Research Service (USDA-ARS), Animal Genomics and Improvement Laboratory, Beltsville, MD 20705, USA; 2 J. Craig Venter Institute, La Jolla, CA, 92037, USA; 3 Department of Electrical and Computer Engineering & TEES-AgriLife Center for Bioinformatics and Genomic Systems Engineering (CBGSE) Texas A&M University, College Station, TX 77843, USA; 4 USDA-ARS, Beltsville Human Nutrition Research Center, Diet, Genomics, and Immunology Laboratory, Beltsville, MD, USA

## Abstract

*Haemonchus contortus* is arguably the most injurious helminth parasite for small ruminants. We characterized the impact of *H. contortus* infection on the caprine abomasal microbiome. Fourteen parasite naive goats were inoculated with 5,000 *H. contortus* infective larvae and followed for 50 days. Six age-matched naïve goats served as uninfected controls. Reduced bodyweight gain and a significant increase in the abosamal pH was observed in infected goats compared to uninfected controls. Infection also increased the bacterial load while reducing the abundance of the Archaea in the abomasum but did not appear to affect microbial diversity. Nevertheless, the infection altered the abundance of approximately 19% of the 432 species-level operational taxonomic units (OTU) detected per sample. A total of 30 taxa displayed a significantly different abundance between control and infected goats. Furthermore, the infection resulted in a distinct difference in the microbiome structure. As many as 8 KEGG pathways were predicted to be significantly affected by infection. In addition, *H. contortus*-induced changes in butyrate producing bacteria could regulate mucosal inflammation and tissue repair. Our results provided insight into physiological consequences of helminth infection in small ruminants and could facilitate the development of novel control strategies to improve animal and human health.

Parasitism has emerged as an important health issue in small ruminants. Parasitic infections cause a substantial decrease in meat, milk, and wool production, and are a leading cause of mortality in sheep^1^ and goats. For example, up to 20% of all deaths in goats are attributed to gastrointestinal parasitism^2^. The helminth parasite *Haemonchus contortus*, a voracious blood feeder residing in the mucosal layer of the abomasum, is arguably the most economically important pathogen in small ruminants grown in tropical and subtropical climates. Clinical manifestations of *H. contortus* infection in goats generally include severe anemia, diarrhea, and dehydration. As a result, infested goats tend to have reduced growth rates, markedly compromised reproduction, and elevated mortality^3^,^4^. Consequently, *H. contortus* infection represents the primary constraint to profitable goat production in many regions of the world^5^.

One of the hallmarks of parasitism by *Teladorsagia circumcincta* and *H. contortus* in small ruminants and *Ostertagia ostertagi* in cattle is increased abomasal pH and hyper-gastrinaemia^6^,^7^,^8^. Infections by adult parasites rapidly alter abomasal secretory activities in these animals. Inhibition of gastric acid production and altered abomasal secretion by the infection are reversible and partly due to parasite-secreted products^6^. Elevated abomasal pH values are closely associated with increased anaerobic bacterial densities in abomasum luminal contents^6^,^7^,^8^. Furthermore, *H. contortus* egg production is strongly correlated with abomasal pH values in sheep^9^, suggesting that interactions among the host, gut microbiome, and the parasite are intricate. Indeed, our previous study in calves infected with *O. ostertagi* demonstrated that parasite infection alters the structure and function of the abomasal microbiome, but a challenge infection of partially immune calves minimally affected the abomasal microbiome and gastric function^7^. However, the effect of helminth infection on the structure and function of the gut microbiome remains largely unknown, especially in small ruminants. In this study, we aimed to characterize changes in microbial composition of the caprine abomasal microbiome induced by *H. contortus.*

## Results

### Haemonchus contortus infection changed the microbial habitat of the caprine abomasum

**Figure 1:**
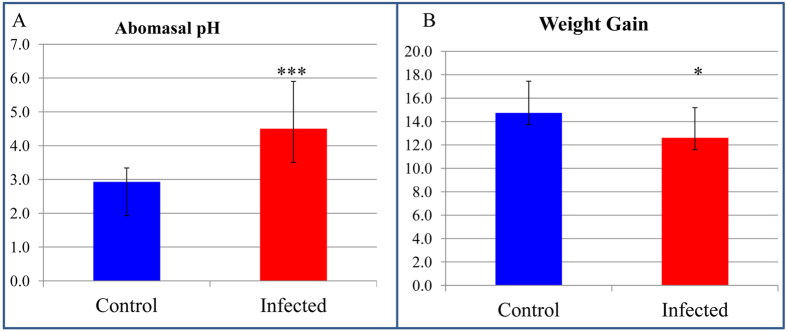
Abomasal pH and bodyweight gain during *Haemonchus contortus* infection in goats. (**A**) Abomasal pH. (**B**) Weight gain (kg). Blue: Uninfected control (*N* = 6); Red: Infected (*N*=14). ***P value < 0.0001; *P value <  0.05.

The primary infection by *H. contortus* in young goats appeared to negatively impact appetite and resulted in a decreased feed intake (Supplementary data). Consequently, infection led to a significantly reduced body weight gain (from 14.7 kg in uninfected control goats to 12.6 kg in infected goats) during the 50-day experiment period (Fig. 1). The infection significantly increased abomasal luminal pH to approximately 4.50 from a normal level of pH 2.93 in control uninfected goats (Fig. 1). Abomasal pH is known to affect parasite egg production when the abomasal pH reaches pH 4.0 to 4.5^9^. Thus, it is conceivable that parasite-induced changes in the microenvironment affect the survival and reproductive capacity of invading parasites.

### Haemonchus colonization did not affect microbial diversity in the caprine abomasum

**Figure 2:**
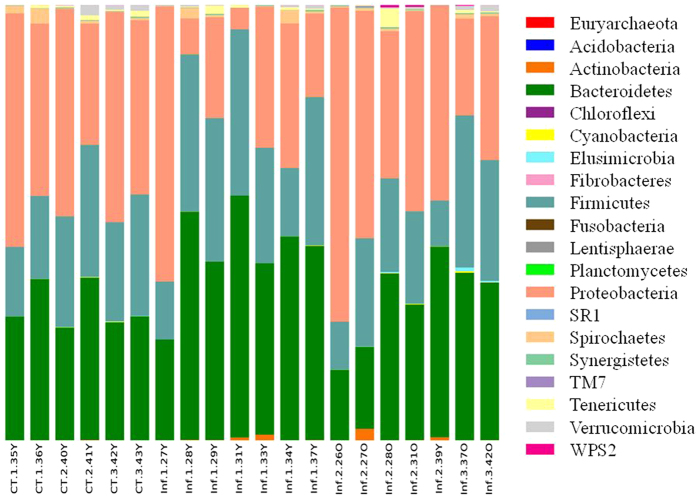
Phylum-level microbial composition in the caprine abomasal microbiome. CT: Uninfected Controls; Inf: Infected.

Microbial diversity in the caprine abomasum was first assessed using the Quantitative Insights Into Microbial Ecology (QIIME) pipeline based on the OTU table (Supplementary data) generated using a “Closed Reference” protocol^10^. A total of 20 phyla were identified (Fig. 2). The most abundant phylum in the caprine abomasum was Proteobacteria, which accounted for approximately 37.3% of all sequences, followed by Bacteroidetes (35.5%), Firmicutes (24.5%), and Spirochaetes (0.8%). Among the 98 families collectively detected, nine families had a relative abundance greater than 1.0%, such as Succinivibrionaceae (36.9%), Prevotellaceae (22.8%), Lachnospiraceae (10.3%), Ruminococcaceae (4.8%), and Veillonellaceae (4.4%). The abomasal microbiome was dominated by 10 families, which accounted for approximately 96% of the sequences. The mean number of OTU (species) in the abomasal microbiome was 432.05 (±72.86, sd). The core microbiome included 43 OTU, which accounted for approximately 71% of the sequences. There were at least 14 OTU with a relative abundance greater than 1.0%, but the caprine abomasal microbiome was dominated by ten most abundant OTU, which accounted for ~59% of all sequences. The core caprine abomasal microbiome included at least three of the 44 named species, such as *Selenomonas ruminantium* (2.3%), *Ruminococcus flavefaciens* (0.2%), and *Clostridium aminophilum* (0.1%).

**Figure 3:**
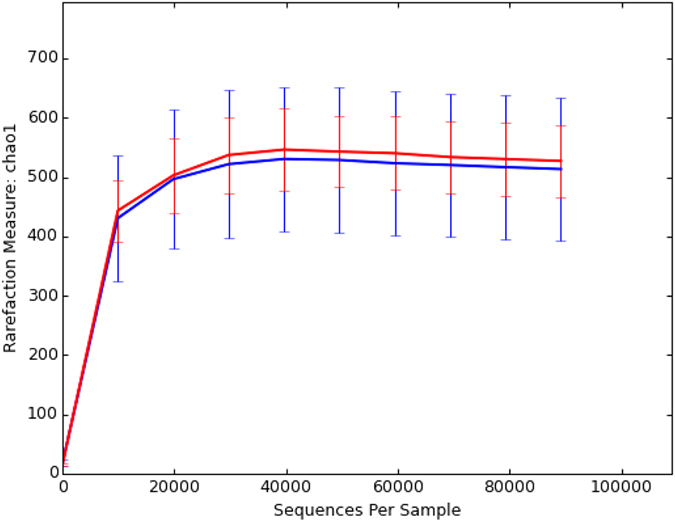
Rarefaction curves based on Chao1 values. Red: Control (*N* = 6); Blue: Infected (*N* = 14). Error bar: SD.

Rarefaction curve analysis suggested that the sequencing depth in this study was adequate (Fig. 3), Common microbial diversity indices, such as Chao1 (Fig. 3), richness, Pielou’s evenness, and Shannon and Simpson indices (Table 1), were evaluated. Our results suggested that *H. contortus* infection in goats did not appear to significantly affect species-level microbial diversity in the abomasal microbiome. For example, the Shannon index (mean ± sd = 4.10 ± 0.28 for control and 4.50 ± 0.88 for infected goats) did not differ significantly (*P* > 0.05 based on a modified *t*-test). In addition, species richness (91.96 ± 9.24 for control and 94.31 ± 18.21 for infected goats) and Phylogenetic Diversity (PD whole tree) (0.36.50 ± 3.45 for control and 36.41 ± 4.91 for infected) were indistinguishable between uninfected control and infected goats (*P* > 0.05).

**Figure 4:**
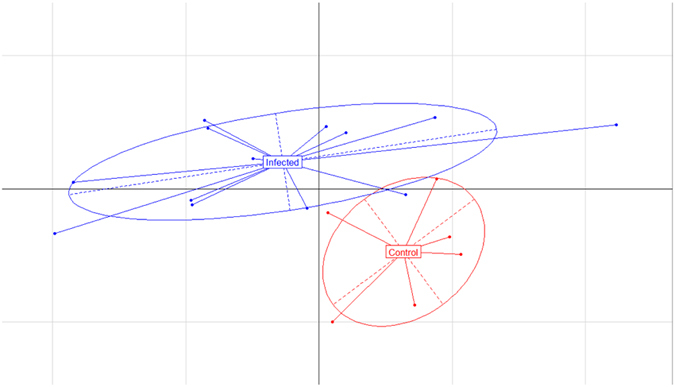
Distinct differences in microbial composition of the caprine abomasal microbiome between the uninfected control and *Haemonchus contortus*-infected goats. Principal component analysis (PCA) was performed using the ade4 package in R based on relative abundance of the 10 most abundant microbial families.

The principal component analysis (PCA) results, however, showed a distinct difference in the microbial composition of the abomasal microbiome between uninfected control and infected goats (Fig. 4).

### Haemonchus infection altered the microbial composition of the abomasal microbiome

**Figure 5:**
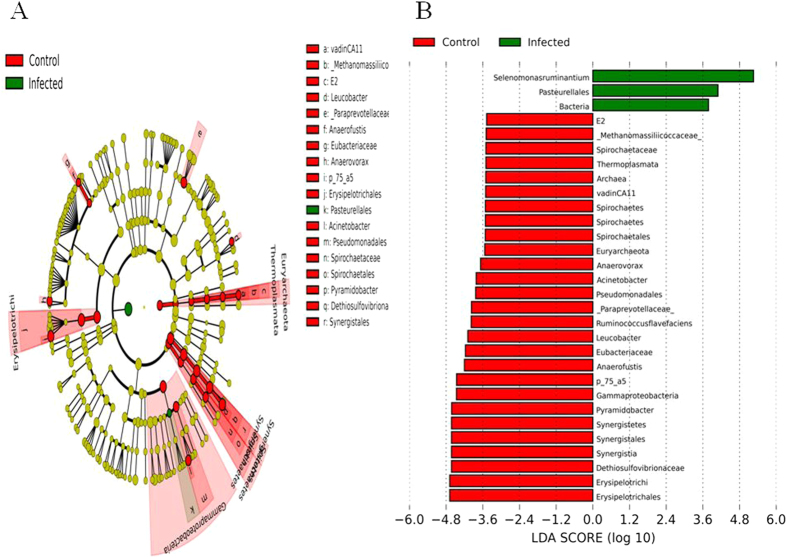
(**A**) a cladogram displaying the taxa with significantly different abundance between the uninfected control and *Haemonchus contortus*-infected goats with an absolute Linear Discriminant Analysis LDA score log10 ≥ 2.0.(**B**) 30 significantly discriminative taxa with absolute LDA score ≥ 2.0.

**Figure 6:**
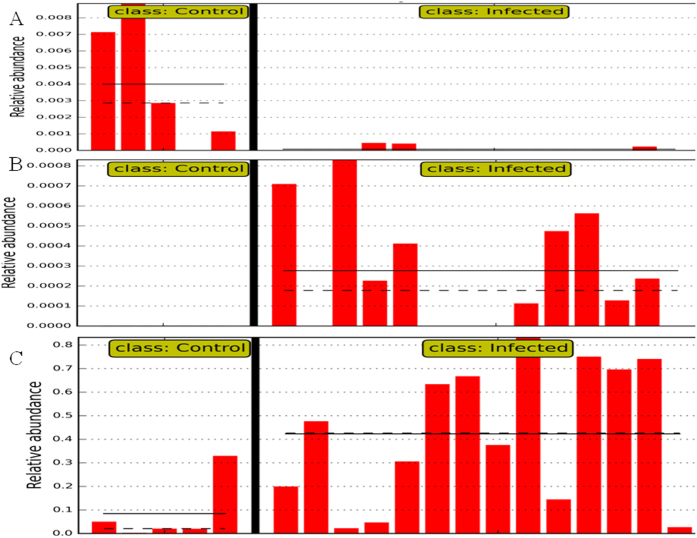
Selected microbial taxa displaying significant differences in relative abundance in the caprine abomasal microbiome between uninfected control and *H.* **contortus-infected goats. X-axis represents the relative abundance of (**A**): Euryarchaeota. **(B)** Pasteurellales. (**C**) *Selenomonas ruminantium*. Y-axis: individual samples. Straight line: mean abundance value of the group. Dotted Line: median of the group.

The difference in the microbial composition (relative abundance) of the abomasal microbiome between goats in the uninfected control and infected groups was compared using the Linear Discriminant Analysis (LDA) Effect Size (LEfSe) algorithm^11^. 81 species-level OTU had a significant difference in relative abundance based on the Wilcoxon non-parametric *t*-test corrected for multiple hypothesis testing (*P* < 0.05). Of them, a total of 30 taxa displayed a significant difference in their abundance between goats in the uninfected control and infected groups at a stringent cutoff value (absolute LDA score log_10_ ≥ 2.0). These taxa were depicted in Fig. 5. The relative abundance of bacteria was significantly higher in goats from the infected group, indicating that the total bacterial load in the abomasum was likely increased by infection. On the other hand, the abundance of the domain Archaea, exclusively from the phylum Euryarchaeota, was significantly higher in goats from the uninfected control group (Fig. 6). The relatively high abundance of Archaea in uninfected control goats was solely due to significantly higher abundance of uncultured species in a candidate family Methanomassiliicoccaceae. At the species level, a total of 44 OTU had a significant difference in their abundance between the two groups (absolute LDA score log_10_ ≥ 2.0). Of them, the 20 most abundant OTU were shown in Table 2. Eight of the 20 OTU contributed to the core abomasal microbiome. For example, *Selenomonas ruminantium* (Fig. 6C) was approximately 37 fold more abundant in the abomasum of infected goats than uninfected control goats (LDA score 4.18).

### Biological pathways and functional categories inferred from the 16S data

Microbial phylogeny and biological function are strongly linked. As a result, gene families and biological pathways can be predicted using 16S rRNA gene sequences. In this study, we analyzed the 16S rRNA gene sequence data to estimate the functional potential of *H. contortus* infection using the Phylogenetic Investigation of Communities by Reconstruction of Unobserved States (PICRUSt) algorithm^12^. After adjusting for copy number variations of 16S rRNA genes, a total of 5,404 KEGG gene families (mean ± sd = 4,283.1 ± 334.7 per sample) were predicted from the OTU table generated using the QIIME closed reference protocol. Of them, six gene families had significantly different abundance between the goats in the control and infected groups by LEfSE (absolute LDA log_10_ scores > 2.0). For example, both α (KEGG#: K00174) and β (K00175) subunits of 2-oxoglutarate ferredoxin oxidoreductase had significantly higher abundance in the infected goats whereas asparagine synthase (K01953), peptide/nickel transport system ATP-binding protein (K02031), simple sugar transport system permease protein (K02057), and methyl-galactoside transport system substrate-binding protein (K10540) were more abundant in the uninfected control group. The OTU contribution analysis suggests that the higher abundance of 2-oxoglutarate ferredoxin oxidoreductase subunits were likely due to the higher incidence of several OTU assigned to the genus *Prevotella* in the infected goats. Likewise, the predominance of simple sugar transport system permease protein (K02057) in goats from the control group may be attributed to the OTU belonging to Clostridia, especially those from the family Lachnospiraceae and the genus *Butyrivibrio*. Among the 30 major OTU contributors to the gene family K02057, 21 belonged to Clostridia, including at least six OTU assigned to *Butyrivibrio*.

**Figure 7:**
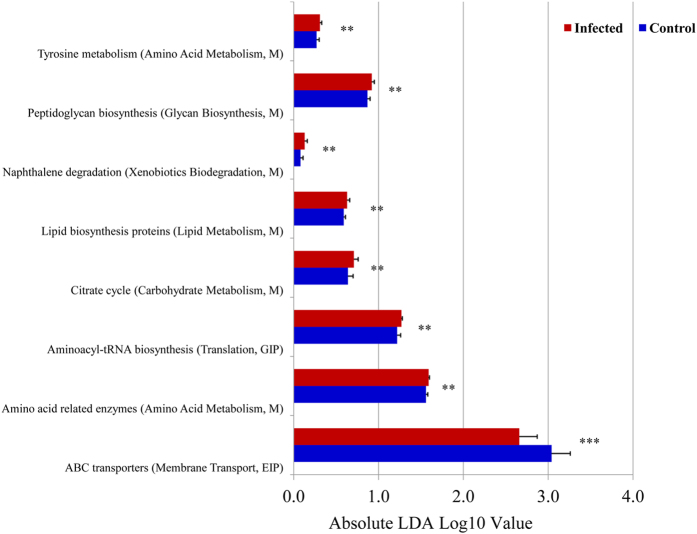
KEGG pathways significantly impacted in the abomasal microbiome during *Haemonchus contortus* infection in goats. Blue: Uninfected control group (*N* = 6); Red: *H. contortus*-infected group (*N* = 14). ***P value < 0.0001; *P value < 0.01.

After classifying >5,400 predicted gene families into higher KEGG functional categories (pathways), LEfSE analysis identified 8 pathways displaying significantly different abundance between goats in the control and infected groups. As shown in Fig. 7, infection may have an effect on a broad range of biological functions, especially on ABC transporters, carbohydrate metabolism (TCA cycle), and amino acid metabolism (such as tyrosine metabolism). Intriguingly, hits assigned to biodegradation of naphthalene, one of the polycyclic aromatic hydrocarbons, were significantly higher in the infected goats, suggesting that *H. contortus* infection may have the potential to modify xenobiotics metabolism in the caprine gut.

## Discussion

The barber’s pole worm *Haemonchus contortus* is the most economically important parasitic nematode of small ruminants and remains the major determinant of profitability and sustainability in sheep and goat production for small farmers worldwide due to increased treatment cost and reduced productivity. Furthermore, the rapid emergence of anthelmintic drug resistant strains in this parasitic species is becoming a serious concern. The parasite elicits a strong immune response; and the first commercial vaccine is available to control the infection^13^. Our recent unpublished results suggested that infection of goats with *H. contortus* activated numerous biological pathways, including cytokine-mediated signaling and type I interferon signaling pathways (*P* < 10^−16^) in the mucosa of the pyloric abomasum. Indeed, immune-mediated pathology rather than direct effects of the parasite itself may be directly responsible for clinical manifestations , such as reduced appetite, weight loss, and diarrhoea^3^.

It has been known that infection with helminth parasites induced a significant change in the structure and function of the host gut microbiome^7^,^14^,^15^. A 21-day infection of pigs with the whipworm *Trichuris suis* significantly altered approximately 13% of genera in the proximal colon microbiome and ~26% of metabolic pathways^14^. Furthermore, the effect of a primary infection with *T. suis* on the porcine proximal colon microbiome appeared to be long-lasting and independent of worm burden^15^. Similar effects on the gastrointestinal microbiome have been observed in hamsters infected with the liver fluke *Opisthorchis viverrini*^16^. A recent study showed that helminth-colonized humans had higher microbial species richness^17^. We performed LEfSe analysis to identify taxa displaying significant differences in abundance between goats infected with *H. contortus* and uninfected control goats. The mean number of species-level OTU identified in the caprine abomasum in this study was approximately 432 while 43 OTU were shared by the abomasal microbiome of all goats tested regardless of infection status. 81 OTU (i.e., ~19% of all OTU identified in individual abomasal microbiomes) were classified as significantly discriminative features before internal Wilcoxon test. Of them, 44 discriminative OTU were detected with an absolute LDA score (log_10_) > 2. However, our results did not show any significant difference in various microbial diversity indices (*P* > 0.05; Table 1), including richness, Pielou’s evenness, and Shannon, Simpson, and Brillouin indices at both the species and genus levels. A recent report also did not detect any effect of infection with the hookworm *Necator americanus* on community structure and microbial diversity in the human fecal microbiome^18^. Diet is known to be the most dominant determinant of the gut microbial composition. In our study, both uninfected control and *H. contortus*-infected goats were fed the same diet and were raised under the same environmental conditions. In contrast, a study of indigenous humans from Malaysia that were infected with helminth parasites showed increased microbial species richness^17^ but the diets of the two human cohorts studied were uncontrolled and could have confounded indicators of microbial diversity.

While *H. contortus* infection in goats did not appear to affect microbial diversity at the site of infection in the abomasum, it did result in a significant alteration to the microbial composition of the abomasal microbiome, including altering the abundance of approximately 19% of all species-level OTU. These changes may be attributed to elevated pH in the microbial habitat because total viable bacteria have been shown to increase 2 to 3 orders of magnitudes to 10^8^–10^9^/ml in the abomasum of sheep as luminal pH rises^19^. Concurrent changes in abomasal anaerobic bacterial densities and pH values due to parasitic infection were also observed in sheep infected with *T. circumcincta*^8^. Our findings showed that *H. contortus* infection in goats tended to increase bacterial abundance with a concomitant reduction in Archaea abundance, which could have an important pathophysiological consequence, especially in host protein metabolism. While depressed appetite resulting from the infection likely reduced nutrient availability to the host and affected weigh gain, increased protein/nitrogen of microbial origin could compensate for this effect. More detailed nutritional determinations are needed to resolve this issue.

*Haemonchus contortus* infection increased the abundance of the genus *Prevotella* as well as the family Prevotellaceae. The mean abundance of *Prevotella* in the abomasal microbiome of the uninfected goats was 16.65% (±5.47; sd) but increased to 25.35% (±11.29; *P* < 0.05) in the infected goats. A total of 148 of the 1,663 OTU detected in this study were positively assigned to *Prevotella*, including *P. ruminicola*. While *Prevotella* abundance varies widely among cows, it appeared to be readily inducible and was the most abundant genus in the rumen^20^,^21^, especially in its liquid fraction^22^,^23^. In humans, the abundance of *Prevotella* was associated with higher carbohydrate or fiber-rich diets^24^. Reduced *Prevotella* incidence was observed in autistic children^25^ as well as in obese Ossabaws pigs^26^. The species consisting of the genus *Prevotella* have versatile biological function, which is generally considered non-cellulolytic but saccharolytic^27^. In ruminants, *Prevotella* species play a crucial role in ruminal protein degradation, especially during oligopeptide breakdown, because they possess a rate-limiting dipeptidyl peptidase type IV (DPP-IV) activity, which is responsible for oligopeptides cleavage^28^. Furthermore, 14 of the 25 major OTU contributors to the gene family 2-oxoglutarate ferredoxin oxidoreductase β (K00175) were assigned to the genus *Prevotella*. 2-oxoglutarate ferredoxin oxidoreductase [EC:1.2.7.3] belongs to a family of oxidoreductases that participate in the citric acid (TCA) cycle, suggesting the *H. contortus*-altered abundance of *Prevotella* affects energy metabolism in the caprine gut microbial ecosystem. During helminth infection in ruminants, feed intake is significantly reduced while endogenous protein loss accelerates due, in part, to the processes that enhance mucin excretion and the synthesis of effector molecules for host immune responses^29^,^30^. As a result, protein availability and quality in the intestine play a larger role as the infection progresses and serves as a main determinant of host nitrogen utilization efficiency^31^. The increased abundance of *Prevotella* in *H. contortus*-infected goats would have functional importance in host protein metabolism. Indeed, *Prevotella* is more abundant in nutrient-inefficient bulls and associated with positive residual feed intake^21^.

The pathophysiological relevance of parasitic infection-induced changes in microbial composition of the gut microbiome is still unclear. It is conceivable that both passive and active mechanisms may be involved. Parasitic infections disrupt microbial habitats. In the caprine abomasum, luminal pH values are significantly higher due to the infection. As a result, survival and proliferation of certain microbial species becomes favored^32^. For example, a substantially elevated level of *Mucispirillum* was observed in the luminal contents of the proximal colon of pigs infected by *T. suis* (whipworms)^14^. *Mucispirillum* species colonize the intestinal mucus and have a predilection for mucosal surfaces. Mucosal disruption induced by invading *T. suis* was likely responsible for dislodging mucus native microbial species to the gut lumen. More importantly, the alteration to the gut microbiome induced by parasitic infection is part of host immune responses^7^ and may represent an adaptive mechanism. In this study, sequences assigned to Pasteurellales (and the family Pasteurellaceae), represented by a group of pathogenic Gram-negative bacteria, were absent in the uninfected control goats but significantly increased in *H. contortus*-infected goats (Fig. 6B). A well studied species from this group is *Pasteurella multocida*, which is associated with the clinical syndromes defining bovine respiratory diseases (BRD) complex, including pneumonia. *P. multocida*-induced BRD symptoms are related to environmental stresses as well as concurrent viral or bacterial infections^33^. Our data provided further evidence that parasitic infections increased the risk of subsequent bacterial infections. Furthermore, as mentioned above, altered *Prevotella* abundance may be involved in the compensation by the host for its impaired protein metabolism. Parasite infection-induced changes in the composition of the gut microbiome could explain special nutritional needs during the infection that include requirements for the sulfur-containing amino acid methionine and other essential amino acids. Furthermore, it has long been known that helminth parasites have evolved abilities to dampen inflammation for their survival in the host^34^. However, the mechanism by which parasites evade host immune surveillance remains elusive. We hypothesize that helminth infection modulates the gut butyrate biosynthesis by altering the abundance of butyrate-producing bacteria. Butyrate is a potent inhibitor of inflammation^35^. In this study, we observed a significant difference between uninfected control and *H. contortus*-infected goats in the relative abundance of the genus *Butyrivibrio,* known to harbor butyrate-producing bacteria. At the species level, at least two OTU positively assigned to the genus *Butyrivibrio,* GreenGene ID# 292105 and #847896, had 84.5 and 4.1 fold higher abundance in goats from the uninfected control than *H. contortus*-infected groups, respectively (LDA scores > 2.0). Motivated by this observation, we plan to examine the effect of parasitic infection on the faction of butyrate-producing bacteria of the gut microbiome in host-parasite models and their potential role in regulating mucosal inflammation.

The abomasum of ruminants shares similar structure and function with the human stomach. Gastric pH is essential for protein digestion and absorption of nutrients like calcium, iron, and vitamin B12, and gastric acidity also plays a critical role in the pathogenesis of diseases, such as abomasal ulceration, abomasitis, abomasal bloating, and gastric tumors. Low acidic environment in the abomasum functions as a potent barrier against bacterial infection and growth. However, uncontrolled proliferation of bacteria also results in several abomasal diseases in ruminants^36^,^37^. Previous studies suggested that the number of microbes in the human stomach was related to luminal pH changes induced by diseases and therapy^38^. An *in vitro* model has been established to simulate the effect of pH on gastric bacteria^32^. Nevertheless, the goat-*H. contortus* infection model could offer a unique opportunity to understand the pathogenesis of human gastric disorders that are affected by changes in pH and the related effects on microbial communities and function.

## Methods

### Animals and parasitology

Twenty male Alpine dairy kid goats were obtained locally within 72 h after birth and were subsequently raised indoors on a concrete floor throughout the experiment. All goats were housed together in the same building separately by pens. The goat kids were fed milk replacer with 25% protein and 28% fat until weaning at approximately two months of age. After weaning, the young goats were fed brome grass hay *ad libitum* and had free access to water. When the goats reached approximately three months of age, 14 randomly selected goats were orally infected with a single dose of 5,000 *H. contortus* infective third-stage larvae (L3) and maintained for 50 days (primary infection). Six goats were not infected and remained parasite naive throughout the experiment and served as uninfected controls. At 50 days post infection (dpi), both infected and uninfected control goats were sacrificed. The live bodyweight of each animal was recorded immediately prior to the primary infection and prior to necropsy at 50 dpi. Fecal egg counts were monitored four times during the experiment, immediately prior to the experimental inoculation, and at 4 and 6 weeks of the primary infection, and immediately prior to necropsy at 50 dpi, as previously described^39^. Adult worms as well as immature larvae from both contents and the tissue of the abomasum were isolated and counted^39^. Abomasal pH values were measured using a hand-held pH meter. The luminal contents of the abomasum were then sampled and snap frozen in liquid nitrogen prior to storage at −80 °C until total DNA was extracted. During the experiment, animals were handled according to a protocol (Protocol #12–025) approved by the BARC Animal Care and Use Committee; and Institutional Animal Care and Use Committee (IACUC) guidelines were strictly followed. The experimental procedures were carried out in accordance with the approved guidelines.

### DNA extraction, 16S rRNA gene amplicon preparation, and sequencing

Total DNA was extracted from the abomasal luminal contents^14^. Briefly, DNA was extracted using a QIAamp DNA stool kit (Qiagen, Valenica, CA) with some modifications. An eight-minute incubation at 95 °C was used to replace the 70 °C lysis recommended in the standard protocol. DNA integrity was verified using a BioAnalyzer 2000 (Agilent, Palo Alto, CA). DNA concentration was then quantified using a QuantiFluor fluorometer (Promega, Madison, WI). The hypervariable V3 -V4 regions (*E. coli* position 341 to 805) of the 16S rRNA gene, which has been frequently targeted for interrogating bacterial communities^40^, were directly amplified from 10 ng of total DNA with PAGE-purified Illumina platform-compatible adaptor oligos that contain features such as sequencing primers, sample-specific barcodes, and 16S PCR primers (forward primer, 341/357F: NNNNCCTACGGGNGGCWGCAG; reverse primer, 805/785R: GACTACHVGGGTATCTAATCC). The PCR reaction included 2.5 units of AccuPrime Taq DNA Polymerase High Fidelity (Invitrogen, Carlsbad, CA) in a 50-μl reaction buffer containing 200 nM primers, 200 nM dNTP, 60 mM Tris-SO_4_, 18 mM (NH4)_2_SO_4_, 2.0 mM MgSO4, 1% glycerol, and 100 ng/ul bovine serum albumin (New England BioLabs, Ipswich, MA). PCR was performed using the following cycling profile: initial denaturing at 95 °C for two min followed by 20 cycles of 95 °C 30 sec, 60 °C 30 s, and 72 °C 6 s. Amplicons were purified using a Agencourt AMPure XP bead kit (Beckman Coulter Genomics, Danvers, MA) and quantified using a BioAnalyzer high-sensitivity DNA chip and a QuantiFluor fluorometer. The purified amplicons from individual samples were pooled in equal mass (molar) ratios. The purified amplicon pool was further spiked with approximately 25% of whole-genome shotgun libraries prepared using an Illumina TruSeq DNA sample prep kit with a compatible adaptor barcode to enhance sequence diversity during the first few cycles of sequencing for better cluster differentiation. The concentration of the final library pool was quantified using a BioAnalyzer High-sensitivity DNA chip kit (Agilent). The library pool was sequenced using an Illumina MiSeq Reagent Kit on an Illumina MiSeq sequencer. Raw sequence data are deposited to the MG-RAST server (http://metagenomics.anl.gov) and available to the public (ProjectID# 13390; MG-RAST ID 4629311.3 to 4629350.3).

### Bioinformatics and data analysis

The data were preprocessed using MiSeq Control Software (MCS) v2.4.1. Raw sequences were first analyzed using FastQC version 0.11.2 to check basic statistics, such as GC%, per base quality score distribution, and sequences flagged as poor quality. The four maximally degenerate bases (“NNNN”) at the most 5′ end of Read#1 of the read pair, which were designed to maximize the diversity during the first four bases of the sequencing run for better identification of unique clusters and improve base-calling accuracy, were then removed. The presence of forward and reverse PCR primers at the 5′ and 3′ ends of each sequence read was scanned; the reads without primers were discarded. Chimeric reads were also removed. Approximately 96.9% of raw reads were retained after these initial quality control steps. The processed pair-end reads were then merged using PandaSeq v2.8 to generate representative complete nucleotide sequences (contigs) using default parameters^41^. The overlapping regions of the pair-end read were first aligned and scored; and reads with low score alignments and high rate of mismatches were discarded. As a result, high-quality contigs can be formed from approximately 92% of raw read pairs (the mean number of contigs per sample ± sd = 208,241.00 ± 51,142.79; *N* = 20). Contig sequences were initially analyzed using BLAT against the SILVA database^42^. Principal component analysis (PCA) was performed using the ade4 package in R as previously described^15^.

QIIME pipeline (v1.9.1) was used to analyze the 16S rRNA gene sequences^10^. A “closed-reference” protocol in the pipeline was used for OTU picking. The default QIIME parameters were used, except that the quality-filtering based on OTU abundance threshold was lowered to 0.0001%. The latest version of GreenGene database (v13.8) was used for taxonomy assignment (greengenes.lbl.gov). PyNAST (v1.2.2) was used for sequence alignment. In addition, the greedy heuristic clustering algorithm CD-HIT-OTU^43^ (v4.5.5) was used to pick OTU for the comparison purpose (Supplementary data). Alpha (α)-diversity at the species-level was estimated using QIIME and Primer programs. Beta diversity was also estimated using PCA in R. The OTU relative abundance values were analyzed using the LEfSe algorithm^11^ to identify taxa and KEGG gene families and/or pathways that display significant differences between two biological conditions. The algorithm first used the non-parametric factorial Kruskal-Wallis sum-rank test to detect taxa with significantly different abundance with respect to the treatment class, followed by unpaired Wilcoxon rank-sum test to detect biological consistency between uninfected and *H. contortus*-infected goats, and then used LDA to estimate the effect size of each differentially abundant feature. Furthermore, PICRUSt (v1.0.0)^12^, a software package designed to predict metagenome functional contents from marker gene (e.g., the 16S rRNA gene) surveys, was used with default parameters to predict gene contents and metagenomic functional information based on the OTU table generated using the closed-reference protocol in QIIME. Briefly, the OTU table was first normalized by dividing each OTU by the known/predicted 16S copy number by using the PICRUSt workflow *normalize_by_copy_number.py*. The gene contents or the abundance of KEGG Orthologs were predicted from the normalized OTU table using the workflow *predict_metagenomes.py*. The predicted metagenome function was further analyzed by collapsing thousands of KEGG Orthologs into higher functional categories (pathways) (*categorize_by_function.py*). In addition, specific OTU contributing to a given function or pathway was identified by using the workflow *metagenome_contributions.py*.

## Additional Information

**How to cite this article**: Li, R. W. *et al.* The effect of helminth infection on the microbial composition and structure of the caprine abomasal microbiome. *Sci. Rep.* **6**, 20606; doi: 10.1038/srep20606 (2016).

## Supplementary Material

Supplementary Information

## Figures and Tables

**Table 1:** Microbial diversity indices in the caprine abomasal miccrobiome.

Diversity Index	Control	Infected	*P* value
Total species (OTU)	424.50 ± 42.56	435.29 ± 83.82	0.7083
Species richness (Margalef)	91.96 ± 9.24	94.31 ± 18.21	0.7070
Species evenness	0.47 ± 0.03	0.51 ± 0.09	0.1317
Chao1	605.28 ± 81.89	628.09 ± 122.50	0.6334
Phylogenetic diversity (PD whole tree)	36.50 ± 3.45	36.41 ± 4.91	0.9633
Brillouin	2.20 ± 0.15	2.41 ± 0.45	0.1287
Shannon	4.10 ± 0.28	4.50 ± 0.88	0.1450
Simpson	0.84 ± 0.04	0.87 ± 0.11	0.4437

**Table 2:** The 20 OTU with significantly different abundance in the abomasal microbiome of infected and parasite-naive control goats.

GreenGene_id	Control	Infected	LDA log10 score	Annotation (Phylum|Class|Order|Family|Genus|Species)
590911	5.4422 ± 3.0784	1.3660 ± 2.9862	4.25	Bacteroidetes|Bacteroidia|Bacteroidales
239386	0.0886 ± 0.1306	3.2822 ± 3.0678	4.18	Firmicutes|Clostridia|Clostridiales|Veillonellaceae|Selenomonas|Selenomonas ruminantium
133732	3.8329 ± 3.8577	0.6993 ± 1.2598	4.25	Firmicutes|Clostridia|Clostridiales|Lachnospiraceae
539073	0.1293 ± 0.1972	1.4637 ± 1.5091	3.79	Bacteroidetes|Bacteroidia|Bacteroidales|S24-7
345464	0.8897 ± 1.0030	0.2748 ± 0.2909	3.53	Firmicutes|Clostridia|Clostridiales|Lachnospiraceae
813220	0.0012 ± 0.0018	0.6061 ± 0.5347	3.41	Firmicutes|Clostridia|Clostridiales|Veillonellaceae|Succiniclasticum
578586	0.0008 ± 0.0009	0.5764 ± 0.6224	3.36	Bacteroidetes|Bacteroidia|Bacteroidales
212832	0.0004 ± 0.0006	0.5039 ± 0.5642	3.30	Bacteroidetes|Bacteroidia|Bacteroidales|Prevotellaceae|Prevotella
558280	0.8318 ± 0.6544	0.1267 ± 0.1639	3.67	Bacteroidetes|Bacteroidia|Bacteroidales|Prevotellaceae|Prevotella
538956	0.5198 ± 0.3754	0.1774 ± 0.3303	3.19	Bacteroidetes|Bacteroidia|Bacteroidales
816483	0.0655 ± 0.0809	0.3274 ± 0.2068	2.97	Bacteroidetes|Bacteroidia|Bacteroidales
655812	0.7137 ± 0.8373	0.0014 ± 0.0022	3.41	Bacteroidetes|Bacteroidia|Bacteroidales|[Paraprevotellaceae]|YRC22
324349	0.0657 ± 0.0630	0.2499 ± 0.2400	3.01	Bacteroidetes|Bacteroidia|Bacteroidales|Prevotellaceae|Prevotella
268684	0.4271 ± 0.4981	0.0039 ± 0.0122	2.98	Tenericutes|Mollicutes|Anaeroplasmatales|Anaeroplasmataceae|Anaeroplasma
355015	0.0357 ± 0.0260	0.1698 ± 0.1441	2.82	Bacteroidetes|Bacteroidia|Bacteroidales|Prevotellaceae|Prevotella
303448	0.0887 ± 0.0534	0.0043 ± 0.0097	2.65	Firmicutes|Clostridia|Clostridiales|Ruminococcaceae|Ruminococcus|Ruminococcus flavefaciens
847896	0.0488 ± 0.0565	0.0119 ± 0.0128	2.40	Firmicutes|Clostridia|Clostridiales|Lachnospiraceae|Butyrivibrio
292105	0.0737 ± 0.1305	0.0009 ± 0.0021	2.71	Firmicutes|Clostridia|Clostridiales|Lachnospiraceae|Butyrivibrio
99436	0.0004 ± 0.0006	0.0200 ± 0.0197	2.02	Firmicutes|Clostridia|Clostridiales|Veillonellaceae|Selenomonas|Selenomonas ruminantium
898468	0.0004 ± 0.0006	0.0000 ± 0.0000	2.77	Actinobacteria|Actinobacteria|Actinomycetales|Microbacteriaceae|Leucobacter
